# Effect of Calcium Supplementation on Blood Lead Levels in Pregnancy: A Randomized Placebo-Controlled Trial

**DOI:** 10.1289/ehp.11868

**Published:** 2008-09-02

**Authors:** Adrienne S. Ettinger, Héctor Lamadrid-Figueroa, Martha M. Téllez-Rojo, Adriana Mercado-García, Karen E. Peterson, Joel Schwartz, Howard Hu, Mauricio Hernández-Avila

**Affiliations:** 1 Harvard School of Public Health, Boston, Massachusetts, USA; 2 University of Michigan School of Public Health, Ann Arbor, Michigan, USA; 3 Mexican National Institute of Public Health, Cuernavaca, Morelos, México; 4 University of Michigan School of Medicine, Ann Arbor, Michigan, USA; 5 Mexican Ministry of Health, Distrito Federal, México

**Keywords:** calcium, diet, lead, pregnancy, randomized trial, supplementation

## Abstract

**Background:**

Prenatal lead exposure is associated with deficits in fetal growth and neurodevelopment. Calcium supplementation may attenuate fetal exposure by inhibiting mobilization of maternal bone lead and/or intestinal absorption of ingested lead.

**Objective:**

Our goal was to evaluate the effect of 1,200 mg dietary calcium supplementation on maternal blood lead levels during pregnancy.

**Methods:**

In a double-blind, randomized, placebo-controlled trial conducted from 2001 through 2003 in Mexico City, we randomly assigned 670 women in their first trimester of pregnancy to ingest calcium (*n* = 334) or placebo (*n* = 336). We followed subjects through pregnancy and evaluated the effect of supplementation on maternal blood lead, using an intent-to-treat analysis by a mixed-effects regression model with random intercept, in 557 participants (83%) who completed follow-up. We then conducted as-treated analyses using similar models stratified by treatment compliance.

**Results:**

Adjusting for baseline lead level, age, trimester of pregnancy, and dietary energy and calcium intake, calcium was associated with an average 11% reduction (0.4 μg/dL) in blood lead level relative to placebo (*p* = 0.004). This reduction was more evident in the second trimester (−14%, *p* < 0.001) than in the third (−8%, *p* = 0.107) and was strongest in women who were most compliant (those who consumed ≥ 75% calcium pills; −24%, *p* < 0.001), had baseline blood lead > 5 μg/dL (−17%, *p* < 0.01), or reported use of lead-glazed ceramics and high bone lead (−31%, *p* < 0.01).

**Conclusion:**

Calcium supplementation was associated with modest reductions in blood lead when administered during pregnancy and may constitute an important secondary prevention effort to reduce circulating maternal lead and, consequently, fetal exposure.

Despite improvements in environmental policies and significant reductions in average U.S. blood lead levels, lead exposure remains a concern for pregnant and lactating women. This is particularly true among certain population subgroups at increased risk, such as women from developing countries and those with occupational exposures [Centers for Disease Control and Prevention (CDC) 2007; Meyer et al. 2003]. In addition, overall declines in environmental sources highlight maternal bone as a long-lived endogenous source of exposure that poses a potential hazard for the developing fetus and breast-feeding infant (Hu and Hernández-Avila 2002). Redistribution of cumulative maternal bone lead stores into the circulation occurs during periods of increased bone resorption, such as pregnancy and lactation (Gulson et al. 2003; Manton et al. 2003; Téllez-Rojo et al. 2004). Prenatal lead exposure has adverse influences on infant birth and neurodevelopmental outcomes across a wide range of exposure (Bellinger 2005; Hu et al. 2006), and maternal bone lead has been shown to be an independent risk factor (Gomaa et al. 2002; Gonzalez-Cossío et al. 1997; Hernández-Avila et al. 2002).

The potential role of nutrition in altering susceptibility to lead exposure and toxicity has long been recognized (Aub et al. 1932; Hu et al. 1995; Mahaffey 1974, 1990). Dietary intake concurrent to exposure is known to have an impact on lead dynamics, and nutrients may interact with lead by binding lead in the gut, competing with lead for absorption, altering intestinal cell avidity for lead, and altering affinity of target tissues for lead (Ballew and Bowman 2001).

Inadequate calcium consumption has been shown to increase lead absorption (Heard and Chamberlain 1982) and retention (Six and Goyer 1970). Lead competes with calcium at calcium-binding sites and may subsequently alter protein function and calcium homeostasis (Sauk and Somerman 1991). Evidence indicates that low dietary calcium and vitamin D are risk factors for elevated bone lead levels (Cheng et al. 1998). Higher milk intake during pregnancy also has been associated with lower maternal and umbilical cord lead levels in postpartum women (Hernández-Avila et al. 1997), suggesting that calcium status may be an important factor in the maternal–fetal transfer of lead across the placenta.

Calcium requirements are increased substantially during pregnancy and lactation in order to meet the needs of the developing fetus and nursing infant for skeletal mineralization and growth (Prentice 2000). Maternal calcium homeostasis is maintained by controlling intestinal calcium absorption, renal calcium excretion, and mobilization of skeletal mineral stores (Kovacs and Kronenberg 1997). The role of dietary calcium and mineral adequacy on skeletal changes of pregnancy and lactation is controversial; however, it is recommended that pregnant and breast-feeding women consume 1,000–1,300 mg calcium per day, depending on their age (Institute of Medicine 1997).

In a randomized, double-blind, placebo-controlled trial of 1,200 mg daily calcium supplementation in lactating women, we have previously shown that calcium supplementation reduced maternal blood lead by 15–20% (Hernández-Avila et al. 2003) and breast milk lead by 5–10% (Ettinger et al. 2006) over the course of lactation. Our objective in the present study was to evaluate the effect of 1,200 mg daily calcium supplementation on maternal blood lead levels during pregnancy, the period of greater relevance for maternal–fetal transfer of lead.

## Materials and Methods

### Study population and design

**Figure 1:**
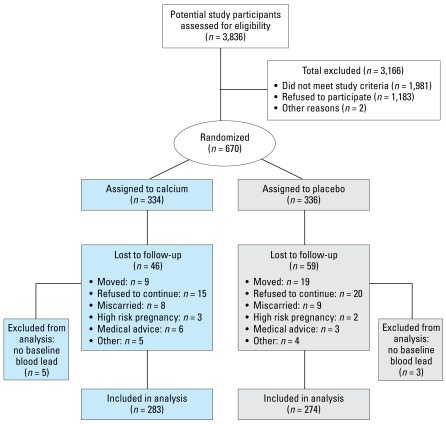
Study sample profile.

We recruited pregnant women from 2001 through 2003 at the Mexican Social Security Institute (Instituto Mexicano del Seguro Social) pre-natal clinics that serve a low- to moderate-income population in Mexico City. We assessed 3,836 women for eligibility, of whom 1,981 did not meet study eligibility criteria (pregnancy of no more than 14 weeks’ gestation; not presenting with a high-risk pregnancy; plans to reside in the metropolitan Mexico City area for ~ 5 years) or had other reasons not being enrolled (*n* = 2). Of the remaining 1,853 eligible women, 670 (36%) agreed to participate and signed the informed consent, and were randomly assigned to receive a daily supplement of 1,200 mg calcium [two 600-mg calcium carbonate tablets (Wyeth Consumer Health Care/Lederle Laboratories, Inc., México City, México) at bedtime; *n* = 334] or placebo (*n* = 336). We assessed blood lead levels, dietary calcium intake, and reported use of lead-glazed ceramics (LGC) at three time points: baseline (first trimester), 6 months (second trimester), and 8 months (third trimester). We assessed compliance by pill count at each follow-up visit. We defined women who had at least one blood lead measurement at 6 or 8 months’ gestation (*n* = 565; 84%) as having completed follow-up. Eight women did not have baseline blood lead levels, yielding a total of 557 subjects (83%) available for inclusion in the final analyses (Figure 1).

The research protocol was approved by the Human Subjects Committee of the National Institutes of Public Health, the Mexican Social Security Institute, the Brigham and Women’s Hospital, and the Harvard School of Public Health and complied with both Mexican and U.S. federal guidelines governing the use of human participants. All participating mothers received a detailed explanation of the study intent and procedures and were advised on identifying and avoiding LGC pottery use during pregnancy before signing the approved written informed consent.

### Blood lead measurement

Blood lead measurements(1.0 μg/dL = 0.0483 μmol/L) were performed using graphite furnace atomic absorption spectrophotometry (Perkin-Elmer model 3000; Norwalk, CT, USA) at the American British Cowdray (ABC) Hospital Trace Metal Laboratory according to a technique described in Miller et al. (1987). The laboratory participates in the CDC blood lead proficiency testing program administered by the Wisconsin State Laboratory of Hygiene (Madison, WI, USA) and maintained acceptable precision and accuracy over the study period.

### Bone lead measurement

At 1 month postpartum (± 5 days), maternal bone lead was estimated by a spot-source cadmium-109 K-X-ray fluorescence (K-XRF) instrument at the research facility at the ABC Hospital. We used two 30-min *in vivo* measurements of each subject’s midtibial shaft (representing cortical bone) and patella (trabecular bone). The physical principles, technical specifications, validation, and use of the K-XRF technique have been described in detail elsewhere (Chettle et al. 2003; Hu et al. 1998). For quality control, we excluded bone lead measurements with uncertainty estimates > 10 and 15 μg lead/g mineral bone for tibia and patella, respectively.

### Dietary intake

We assessed maternal dietary intake in each trimester of pregnancy using a semiquantitative food frequency questionnaire designed to estimate usual dietary intake over the prior month. We based the questionnaire on the semiquantitative food frequency questionnaires and validation methodology used in the Harvard Nurses’ Health Study and Health Professionals’ Follow-up Study (Willett et al. 1985, 1987). We translated the questionnaire and validated it for use specifically for the Mexican Spanish-speaking adult population (Hernández-Avila et al. 1998).

### Statistical analysis

We compared baseline characteristics of participants between the calcium and placebo groups using Wilcoxon ranksum (Mann–Whitney *U*-test) two-sample test of equality or Student’s *t*-test, as appropriate. We performed a similar comparison between those included in the analyses and those lost to follow-up.

We evaluated the effect of calcium supplement on blood lead concentration using an intent-to-treat analysis by means of a mixed-effects regression model with a random intercept for each subject. This approach takes into account the within-subject correlation structure attributed to the repeated measurements, yielding valid standard errors of the effect estimates. Blood lead concentrations in the second and third trimester of pregnancy were the outcome variables; however, we used models featuring natural-log– transformed blood lead because this parameterization provided the best fit. In order not to exclude very low blood lead concentrations from the analysis, we substituted 27 blood lead measure ments (1.6% of the total) below the limit of detection (1 μg/dL) with random numbers following a uniform distribution between 0 and 1. We adjusted models for the following baseline variables: first trimester log-transformed blood lead concentration, maternal age (years), treatment group, daily calcium (grams per day) and energy intake (kilocalories per day), and trimester of pregnancy.

To assess the overall intent-to-treat effect of calcium supplementation on blood lead concentrations throughout the last two trimesters of pregnancy, we fitted the following model:


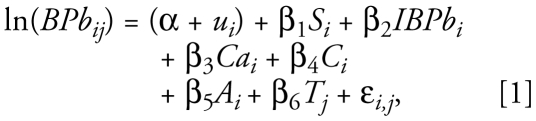


where ln(BPb*_i,j_*) is the log*_e_*-transformed blood lead concentration for subject *i* at trimester *j*, α + *u_i_* denotes the random intercept where *u_i_* represents the error term associated to the *i*th subject [*i* = (1, 2, . . . , *n*); *u_i_* ~ *n*(0, σ^2^*u*)], *S_i_* is a dummy variable that indicates treatment assignment, lnBPb*_i_* is the initial (baseline) natural log-transformed blood lead concentration of the *i*th subject, *T_j_* is the *j*th trimester of pregnancy [*j* = (2,3)], *C_i_* is the baseline daily energy intake, *Ca_i_* is the baseline daily calcium dietary intake, *A_i_* is age, and ɛ*_i_*_,_*_j_* denotes the random variation [ɛ_i,j_ ~ *n*(0, σ^2^)]. The overall treatment effect estimate is the coefficient β_1_.

We fitted a second model to estimate the treatment effect at each trimester:


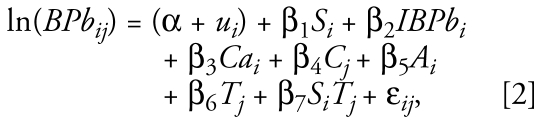


where *S_i_T_j_* denotes the interaction term between blood lead levels and trimester of pregnancy. The treatment effect estimate in the second trimester is the coefficient β_1_, and the effect in the third trimester is (β_1_ + β_7_).

We used a secondary dose–response study to further assess the effectiveness of supplementation. We assessed compliance by pill count at each visit and analyzed it as proportion of expected pills used between baseline (first trimester) and end of follow-up (8 months’ gestation). We defined treatment compliance group in three ways: ≥ 50% of pills consumed, ≥ 67% of pills consumed, and ≥ 75% of pills consumed. To try to disentangle the effect of calcium supplementation on bone lead mobilization versus gastrointestinal absorption, we developed models with an interaction model for postpartum bone lead levels and reported use of LGC. The rationale for fitting this model was that the effect of the supplement may have been larger in those who had larger bone lead concentration and/ or in those who used LGC. We generated a new dummy variable designating high and low patella bone lead levels dichotomized at the median (5.6 μg/g) and created a two-way interaction term with LGC use (yes/no). We did not include a three-way interaction because we found no reason to think that the magnitude of the effect of bone lead concentrations, and thus bone lead mobilization rates, on blood lead would depend on the use of LGC. We fitted the following model:


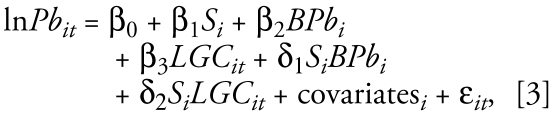


where Pb*_it_* is blood lead concentration for the *i*th subject at the *t*th trimester, *S_i_* is the supplementation group, *BPb_i_* is the first available postpartum bone lead measurement, *LGC_it_* is current use of LGC in the *i*th subject at the *t*th trimester, and δ_1_ represents the difference in the effect of supplementation between the high and low bone Pb concentration groups, and δ_2_ represents the difference in the effect of supplementation between the current use/ not use of LGC groups. Covariates are baseline blood lead level, baseline daily calcium dietary intake, baseline daily energy intake, age, and trimester of pregnancy. Because we were trying to disentangle biologic mechanisms, we restricted these models to those who with ≥ 75% compliance.

Finally, to account for possible heterogeneity of treatment effects according to initial blood lead levels, we also performed analysis by baseline blood lead group (< 5 μg/dL vs. ≥ 5 μg/dL) using an intent-to-treat analysis and then among only those women with ≥ 50% compliance.

We performed all statistical analyses using Stata for Windows (version 9.0; StataCorp LP, College Station, TX, USA).

## Results

We randomized 670 eligible women to receive calcium supplementation (*n* = 334) or placebo (*n* = 336) (Figure 1). Baseline characteristics were largely similar for both the calcium and placebo groups. Mean maternal age was 1 year higher in the control group (26.9 years) than in the calcium group (25.9 years; *p* = 0.02) (Table 1). Approximately 35% of women reported current use of traditional LGC for storing, preparing, or serving food; however, we found no significant differences by treatment group. Dietary calcium intake, also not significantly different between the two groups, was about 900 mg/day on average. Geometric mean (and geometric standard deviation) prerandomization blood lead levels were 3.8 (2.0) and 4.1 (2.0) μg/dL for the calcium and placebo groups, respectively (*p* = 0.05).

A total of 565 women (84%) completed follow-up. Comparing the group that completed follow-up (placebo *n* = 277; calcium *n* = 288) with those lost to follow-up [placebo *n* = 59 (18%); calcium *n* = 46 (14%)], we found no significant differences by treatment group assignment (*p* = 0.18). Those women who remained in the study reported higher daily energy intake (*p* < 0.01) and higher use of LGC (*p* = 0.04) at baseline. Those women who completed follow-up reported higher current use of LGC (36%) than those who did not complete follow-up (26%); among those completing follow-up, however, we found no significant differences in reported LGC use by treatment group.

In the intent-to-treat analysis (*n* = 557), calcium supplementation was associated with an overall average reduction of 11% in maternal blood lead concentrations relative to placebo (*p* = 0.004) (Table 2). In a secondary analysis, this reduction was more evident in the second trimester (14% reduction, *p* < 0.001) than in the third trimester (8% reduction, *p* = 0.107). These results did not change when we controlled for hematocrit level (data not shown).

**Figure 2:**
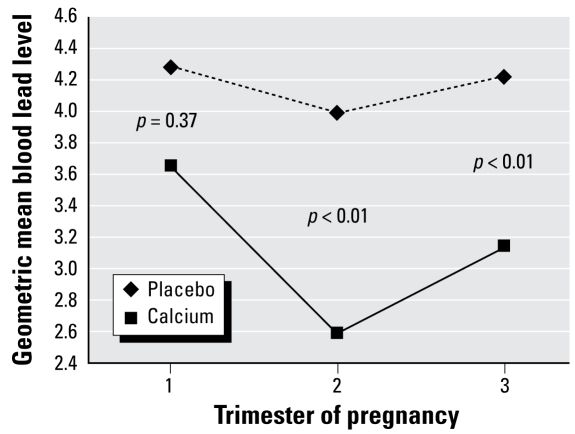
Effect of calcium supplementation on maternal blood lead at each trimester during pregnancy among the high-compliance group (≥ 75% of pills by pill count, adjusting for baseline blood lead, age, dietary calcium intake, and daily energy intake.

When we assessed the dose–response effect of calcium supplementation for women “as treated” (*n* = 557) using models stratified by treatment compliance, we saw a clear dose–response effect of calcium on blood lead concentration (Table 3). Among those women who consumed ≥ 50% of pills, calcium was associated on average with a 15% reduction in blood lead levels compared with those taking placebo (*p* < 0.001). This increased to 19% (*p* < 0.001) and 24% (*p* < 0.001) for those who consumed ≥ 67% of pills and ≥ 75% of pills, respectively (*p* for trend < 0.001). Figure 2 shows the effects of calcium and placebo on maternal blood lead over time among the high-compliance group.

Among the low-compliance group (< 50% of pills consumed), blood lead was higher in the calcium-supplemented group, suggesting that these women were somehow different from the low compliers receiving placebo. In fact, in the group that completed follow-up (*n* = 565), those with low compliance reported higher current use of LGC in the calcium group (35%) compared with placebo (27%), which might explain the apparent increase in blood lead among the low-compliance group. We found no significant differences in reported LGC use among the high-compliance group.

**Figure 3:**
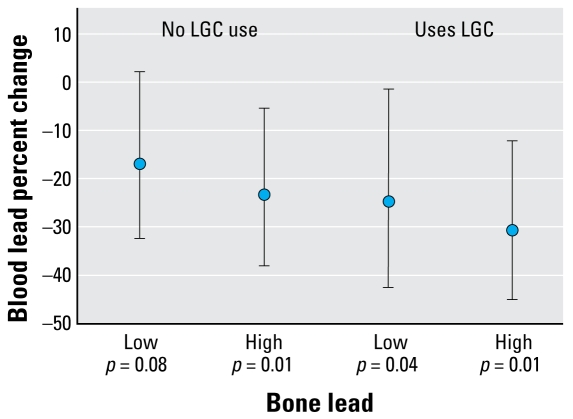
Blood lead proportional reduction estimates due to calcium supplementation (and 95% CIs), stratified by use of LGC (yes/no) and bone lead level (high/low) among the high-compliance group (≥ 75% of pills by pill count, adjusting for baseline blood lead, age, dietary calcium intake, daily energy intake, and trimester of pregnancy).

Figure 3 shows the proportional reduction [and 95% confidence intervals (CIs)] in blood lead due to calcium supplementation, stratified by use of LGC and patella lead level, among the high-compliance group. Among women consuming ≥ 75% of pills, those with high patella bone lead experienced greater reductions than those women with lower bone lead levels, corresponding to a 23% reduction (*p* = 0.01) for those with no reported use of LGC and a 31% reduction (*p* < 0.01) for those who reported use of LGC. In this subset of most compliant women with high patella bone lead (> 5 μg/g) and reported use of LGC, the effect corresponds to an average blood lead reduction of 1.95 μg/dL (95% CI, −0.78 to −2.87).

We repeated the analysis by baseline blood lead group (< 5 μg/dL vs. ≥ 5 μg/dL) using intent-to-treat and as-treated analyses among only those women with compliance ≥50% of pills consumed (Table 4). The effects of calcium appeared stronger in the group with higher blood lead at baseline (17% reduction), compared with those with baseline blood lead levels < 5 μg/dL (7% reduction). However, when we restricted the analysis to those women who were more compliant, the reductions were similar between the women with higher (≥ 5 μg/dL = 17%) and lower (< 5 μg/dL = 14%) blood lead at baseline. Among those women with low compliance (< 50% of pills; *n* = 82), those with low baseline blood lead (< 5 μg/dL) appeared to experience a paradoxical effect of calcium on blood lead levels (an increase of 34%). Those who started the study with higher blood lead (≥ 5 μg/dL) showed the same average effects of treatment (17% reduction), although not statistically significant. Further analysis revealed, however, that the reported use of LGC in low compliers was higher among the calcium group (35%) than in the placebo group (27%), which may account for the apparent differences in treatment effect (7% vs. 17% reduction) observed in the intent-to-treat analysis by baseline blood lead.

## Discussion

In this randomized control trial, calcium supplementation (1,200 mg) was associated with modest reductions in blood lead levels when administered during pregnancy. These effects were clearly stronger with increasing compliance, with a 24% average reduction in the most compliant women, and strongest in those with baseline blood lead level > 5 μg/dL (17% average reduction). In the subset of most compliant women with high patella bone lead (> 5 μg/g) and reported use of LGC, we found the greatest reduction in blood lead of 31%, which corresponds to an average reduction of 1.95 μg/dL (95% CI, −0.78 to −2.87).

These results are consistent with our previously published randomized trial, which showed that dietary calcium supplementation among postpartum women reduced maternal blood lead by 15–20% over the course of lactation (Hernández-Avila et al. 2003). In that study, the effect among women who were compliant with supplement use (≥ 50% of pills consumed) and had high bone lead (patella lead > 5 μg lead/g bone mineral) was an estimated reduction in mean blood lead of 1.16 μg/dL (95% CI, −2.08 to −0.23).

These results are also consistent with the results of a study by Gulson et al. (2004) of blood lead isotopic ratios during pregnancy among women who had recently immigrated to Australia. The authors found that compared with an earlier group of such women they had studied who had calcium-deficient diets, calcium-replete women had a rise in blood lead levels during pregnancy (with an isotopic fingerprint suggesting the lead came from bone) that occurred later in pregnancy and of a smaller magnitude. Although the use of lead isotopic ratios by Gulson et al. (2004) provided very rigorous and precise methodology to their work, the interpretation with respect to calcium supplementation is limited by the small number of women (< 20) in their cases series [and thus limited statistical power to detect an association (Altman and Bland 1995)] and issues of comparability (e.g., the calcium-deficient women were studied at an earlier time and came from Central Europe, whereas the calcium-replete women were studied at a later time, came from Asia, and were otherwise not matched), making the results of our randomized placebo-controlled trial of particular interest.

The effect of calcium may be exerted, at least in part, by decreasing bone resorption and the consequent mobilization of maternal bone lead stores. In a case–crossover trial of calcium supplementation during the third trimester of pregnancy, we have previously shown that maternal bone resorption, as reflected by urinary cross-linked *N*-telopeptide, was reduced by an average of 13.6 nM bone collagen equivalents/mM creatinine (14%) compared with placebo (Janakiraman et al. 2003), indicating that calcium supplementation can suppress maternal bone mobilization.

The effects of calcium may also be attributed to decreasing the intestinal absorption of lead and/or increasing the excretion of lead from circulation. In the present study, we did not have prepregnancy bone lead levels, and Mexican laws forbidding potential radiation exposure during pregnancy did not allow us to obtain bone lead measurements during pregnancy. However, our observation in the stratum of women with no reported LGC use—that the calcium effect is greater in those with high bone lead—suggests that, in this population, the effect may have been exerted mainly through inhibiting bone resorption.

Average baseline dietary calcium intake for women in our trials of Mexican women was less than the U.S. recommended dietary intake of 1,000–1,300 mg/day for pregnant and lactating women (Institute of Medicine 1997). Levels of dietary calcium intake in our studies were, however, consistent with those reported in the Mexican National Nutrition Survey (Barquera et al. 2003) and in a nationally representative sample of U.S. women of child-bearing age (Lee et al. 2005). Hertz-Picciotto et al. (2000) followed 195 women over the course of pregnancy and found a U-shaped pattern of maternal blood lead concentration across pregnancy. The late pregnancy increases were steeper among women with low dietary calcium intake in both the low and high age groups, suggesting that lead redistribution may be more pronounced among pregnant women in calcium-deficient states. It is possible that high amounts of calcium are needed to counterbalance the nutritional needs of the developing fetus (Johnson 2001). Other genetic, hormonal, or lifestyle factors may also be involved (Ettinger et al. 2007).

Nonetheless, dietary calcium intake likely plays a limited, but still important, role in suppressing mobilization of lead from maternal bone and/or decreasing gastrointestinal absorption of ingested lead, thereby decreasing the risk of fetal and infant exposure. Calcium supplementation during pregnancy may also reduce the risk of hypertensive disorders of pregnancy (Hofmeyr et al. 2007) that may also arise secondary to lead exposure (Rothenberg et al. 2002; Sowers et al. 2002) (and thus conferring additional negative effects of lead for both mother and fetus and a potential benefit of calcium supplementation). The risks posed by calcium supplementation at levels approximating recommended daily intakes in this population are negligible. We therefore conclude that dietary supplementation of calcium intake should be considered as a cost-effective means for lowering transgenerational fetal lead exposure. This is particularly important in populations where dietary calcium intake is low. Because bone lead has a half-life of years to decades, women and their infants will continue to be at risk for exposure long after environmental sources of lead have been abated.

## Figures and Tables

**Table 1:** Baseline characteristics of participants, by treatment assignment and follow-up status.

	Treatment assignment		Follow-up status	
Variable	Calcium (*n* = 334)	Placebo (*n* = 336)	Completed^a^ (*n* = 565)	Lost (*n* = 105)
Age (years)	26.9 ± 5.6	25.9 ± 5.3^b^	26.5 ± 5.5	26.2 ± 5.4
Education (years)	10.8 ± 2.9	10.6 ± 2.9	10.7 ± 2.9	10.7 ± 2.8
No. of pregnancies	2.0 ± 1.0	2.1 ± 1.1	2.1 ± 1.1	2.0 ± 0.9
Weight (kg)	61.9 ± 10.7	61.5 ± 10.0	61.6 ± 10.2	62.2 ± 11.0
Height (cm)	154.4 ± 5.6	154.4 ± 5.9	154.3 ± 5.8	155.0 ± 5.8
Dietary calcium intake (g/day)	0.92 ± 0.35	0.89 ± 0.40	0.90 ± 0.38	0.94 ± 0.34
Total energy intake (kcal/day)	2,213 ± 632	2,157 ± 675	2,155 ± 642	2,347 ± 699^b^
Current use of LGC [no. (%)]	117 (35%)	115 (34%)	205 (36%)	27 (26%)^b^
Blood lead (μg/dL)^c^	3.8 (2.0)	4.1 (2.0)	3.8 (2.0)	4.5 (1.9)

Values are mean ± SD except where noted.

a Completed follow-up defined as having at least one follow-up blood lead level at 6 or 8 months of pregnancy.

b *p* < 0.05 Student’s *t*-test or Wilcoxon rank-sum (Mann–Whitney *U*-test) two-sample test of equality for difference in means or chi-square test, as appropriate.

c Geometric mean (geometric standard deviation) blood lead levels.

**Table 2:** Effect of calcium supplementation on blood lead concentration (log-transformed) (*n* = 557; intent-to-treat analysis).

Variable	β-Coefficient	*p*-Value	95% CI
Treatment (calcium = 1; placebo = 0)	−0.117	0.004	(−0.196 to −0.038)
Blood lead at baseline (μg/dL)^a^	0.674	< 0.001	(0.165 to 0.732)
Age (years)	0.007	0.051	(−0.00003 to 0.014)
Dietary calcium intake at baseline (g/day)	−0.024	0.751	(−0.169 to 0.122)
Total energy intake at baseline (1,000 kcal/day)	−0.010	0.813	(−0.096 to 0.075)
Trimester of pregnancy (time: 1 = third; 0 = second)	0.119	< 0.001	(0.073 to 0.166)

a Log-transformed outcome variable, thus estimate of treatment effect: 1 − *e*^−0.117^ = −11% (95% CI, −17.8% to −3.7%).

**Table 3:** Effect of calcium supplementation^a^ by treatment compliance.^b^

		Average (Overall)			Second trimester			Third trimester		
Compliance	No.	β	% Δ^c^	*p*-Value	β	%Δ^c^	*p*-Value	β	%Δ^c^	*p*-Value
All	557	−0.12	−11%	0.004	−0.15	−14%	0.001	−0.08	−8%	0.107
< 50%	82	0.18	20%	0.115	0.30	35%	0.024	0.09	9%	0.497
≤ 50%	475	−0.17	−15%	< 0.001	−0.22	−20%	< 0.001	−0.11	−10%	0.037
≤ 67%	357	−0.20	−19%	< 0.001	−0.28	−24%	< 0.001	−0.12	−11%	0.054
≤ 75%	241	−0.27	−24%	< 0.001	−0.32	−27%	< 0.001	−0.21	−19%	0.004

a Adjusted for baseline blood lead level, maternal age, dietary calcium intake at baseline, daily energy intake at baseline, treatment group, and trimester of pregnancy.

b We assessed compliance by pill count at each visit and analyzed it as proportion of expected pills used between baseline (first trimester) and end of follow-up (8 months’ gestation).

c Percent change; log-transformed outcome variable, thus estimate of treatment effect: 1 − e^−β^.

**Table 4:** Effect of calcium supplementation^a^ by baseline blood lead level.

Baseline blood lead level	No. (calcium/placebo)	β-Coefficient	%Δ^b^	*p*-Value
Among all women with follow-up (intent-to-treat analysis)				
< 5 μg/dL	349 (183/166)	−0.07	−7%	0.08
≥ 5 μg/dL	208 (100/108)	−0.19	−17%	0.003
Among those women with compliance ≥ 50%^c^ (as-treated analysis, among high compliers)				
< 5 μg/dL	292 (162/130)	−0.15	−14%	0.01
≥5 μg/dL	183 (87/96)	−0.19	−17%	0.004
Among those women with compliance < 50%^c^ (as-treated analysis, among low compliers)				
< 5 μg/dL	57 (21/36)	0.29	34%	0.02
≥5 μg/dL	25 (13/12)	−0.18	−17%	0.49

a Adjusting for baseline blood lead level (log-transformed), maternal age, dietary calcium intake at baseline, daily energy intake at baseline, treatment group, and trimester of pregnancy.

b Percent change; log-transformed outcome variable, thus estimate of treatment effect: 1 − *e*^−β^.

c We assessed compliance by pill count at each visit and analyzed as proportion of expected pills used between baseline (first trimester) and end of follow-up (8 months’ gestation).

## References

[b1-ehp-117-26] Altman DG, Bland JM (1995). Absence of evidence is not evidence of absence. BMJ.

[b2-ehp-117-26] Aub JC, Robb GP, Rossmeisl E (1932). Significance of bone trabeculae in the treatment of lead poisoning: lead studies XVII. Am J Public Health Nations Health.

[b3-ehp-117-26] Ballew C, Bowman B (2001). Recommending calcium to reduce lead toxicity in children: a critical review. Nutr Rev.

[b4-ehp-117-26] Barquera S, Rivera JA, Espinosa-Montero J, Safdie M, Campirano F, Monterrubio EA (2003). Energy and nutrient consumption in Mexican women 12–49 years of age: analysis of the National Nutrition Survey 1999. Salud Publica Mex.

[b5-ehp-117-26] Bellinger DC (2005). Teratogen update: lead and pregnancy. Birth Defects Res A Clin Mol Teratol.

[b6-ehp-117-26] CDC (Centers for Disease Control and Prevention) (2007). Lead exposure among females of childbearing age—United States, 2004. MMWR Morb Mortal Wkly Rep.

[b7-ehp-117-26] Cheng Y, Willett WC, Schwartz J, Sparrow D, Weiss S, Hu H (1998). Relation of nutrition to bone lead and blood lead levels in middle-aged to elderly men. The Normative Aging Study. Am J Epidemiol.

[b8-ehp-117-26] Chettle DR, Arnold ML, Aro AC, Fleming DE, Kondrashov VS, McNeill FE (2003). An agreed statement on calculating lead concentration and uncertainty in XRF *in vivo* bone lead analysis. Appl Radiat Isot.

[b9-ehp-117-26] Ettinger AS, Hu H, Hernández-Avila M (2007). Dietary calcium supplementation to lower blood lead levels in pregnancy and lactation. J Nutr Biochem.

[b10-ehp-117-26] Ettinger AS, Téllez-Rojo MM, Amarasiriwardena C, Peterson KE, Schwartz J, Aro A (2006). Influence of maternal bone lead burden and calcium intake on levels of lead in breast milk over the course of lactation. Am J Epidemiol.

[b11-ehp-117-26] Gomaa A, Hu H, Bellinger D, Schwartz J, Tsaih SW, Gonzalez-Cossío T (2002). Maternal bone lead as an independent risk factor for fetal neurotoxicity: a prospective study. Pediatrics.

[b12-ehp-117-26] Gonzalez-Cossío T, Peterson KE, Sanin LH, Fishbein E, Palazuelos E, Aro A (1997). Decrease in birth weight in relation to maternal bone-lead burden. Pediatrics.

[b13-ehp-117-26] Gulson BL, Mizon KJ, Korsch MJ, Palmer JM, Donnelly JB (2003). Mobilization of lead from human bone tissue during pregnancy and lactation—a summary of long-term research. Sci Total Environ.

[b14-ehp-117-26] Gulson BL, Mizon KJ, Palmer JM, Korsch MJ, Taylor AJ, Mahaffey KR (2004). Blood lead changes during pregnancy and postpartum with calcium supplementation. Environ Health Perspect.

[b15-ehp-117-26] Heard MJ, Chamberlain AC (1982). Effect of minerals and food on uptake of lead from the gastrointestinal tract in humans. Hum Toxicol.

[b16-ehp-117-26] Hernández-Avila M, Gonzalez-Cossío T, Hernández-Avila JE, Romieu I, Peterson KE, Aro A (2003). Dietary calcium supplements to lower blood lead levels in lactating women: a randomized placebo-controlled trial. Epidemiology.

[b17-ehp-117-26] Hernández-Avila M, Peterson KE, Gonzalez-Cossío T, Sanin LH, Aro A, Schnaas L (2002). Effect of maternal bone lead on length and head circumference of newborns and 1-month-old infants. Arch Environ Health.

[b18-ehp-117-26] Hernández-Avila M, Romieu I, Parra S, Hernández-Avila J, Madrigal H, Willett W (1998). Validity and reproducibility of a food frequency questionnaire to assess dietary intake of women living in Mexico City. Salud Publica Mex.

[b19-ehp-117-26] Hernández-Avila M, Sanin LH, Romieu I, Palazuelos E, Tapia-Conyer R, Olaiz G (1997). Higher milk intake during pregnancy is associated with lower maternal and umbilical cord lead levels in postpartum women. Environ Res.

[b20-ehp-117-26] Hertz-Picciotto I, Schramm M, Watt-Morse M, Chantala K, Anderson J, Osterloh J (2000). Patterns and determinants of blood lead during pregnancy. Am J Epidemiol.

[b21-ehp-117-26] Hofmeyr GJ, Duley L, Atallah A (2007). Dietary calcium supplementation for prevention of pre-eclampsia and related problems: a systematic review and commentary. BJOG.

[b22-ehp-117-26] Hu H, Hernández-Avila M (2002). Invited commentary: lead, bones, women, and pregnancy—the poison within?. Am J Epidemiol.

[b23-ehp-117-26] Hu H, Kotha S, Brennan T (1995). The role of nutrition in mitigating environmental insults: policy and ethical issues. Environ Health Perspect.

[b24-ehp-117-26] Hu H, Rabinowitz M, Smith D (1998). Bone lead as a biological marker in epidemiologic studies of chronic toxicity: conceptual paradigms. Environ Health Perspect.

[b25-ehp-117-26] Hu H, Téllez-Rojo MM, Bellinger D, Smith D, Ettinger AS, Lamadrid-Figueroa H (2006). Fetal lead exposure at each stage of pregnancy as a predictor of infant mental development. Environ Health Perspect.

[b26-ehp-117-26] Institute of Medicine (1997). Dietary Reference Intakes for Calcium, Phosphorus, Magnesium, Vitamin D and Fluoride.

[b27-ehp-117-26] Janakiraman V, Ettinger A, Mercado-García A, Hu H, Hernández-Avila M (2003). Calcium supplements and bone resorption in pregnancy: a randomized crossover trial. Am J Prev Med.

[b28-ehp-117-26] Johnson MA (2001). High calcium intake blunts pregnancy-induced increases in maternal blood lead. Nutr Rev.

[b29-ehp-117-26] Kovacs CS, Kronenberg HM (1997). Maternal-fetal calcium and bone metabolism during pregnancy, puerperium, and lactation. Endocr Rev.

[b30-ehp-117-26] Lee MG, Chun OK, Song WO (2005). Determinants of the blood lead level of US women of reproductive age. J Am Coll Nutr.

[b31-ehp-117-26] Mahaffey KR (1974). Nutritional factors and susceptibility to lead toxicity. Environ Health Perspect.

[b32-ehp-117-26] Mahaffey KR (1990). Environmental lead toxicity: nutrition as a component of intervention. Environ Health Perspect.

[b33-ehp-117-26] Manton WI, Angle CR, Stanek KL, Kuntzelman D, Reese YR, Kuehnemann TJ (2003). Release of lead from bone in pregnancy and lactation. Environ Res.

[b34-ehp-117-26] Meyer PA, McGeehin MA, Falk H (2003). A global approach to childhood lead poisoning prevention. Int J Hyg Environ Health.

[b35-ehp-117-26] Miller DT, Paschal DC, Gunter EW, Stroud PE, D’Angelo J (1987). Determination of lead in blood using electrothermal atomisation atomic absorption spectrometry with a L’vov platform and matrix modifier. Analyst.

[b36-ehp-117-26] Prentice A (2000). Calcium in pregnancy and lactation. Annu Rev Nutr.

[b37-ehp-117-26] Rothenberg SJ, Kondrashov V, Manalo M, Jiang J, Cuellar R, Garcia M (2002). Increases in hypertension and blood pressure during pregnancy with increased bone lead levels. Am J Epidemiol.

[b38-ehp-117-26] Sauk JJ, Somerman MJ (1991). Physiology of bone: mineral compartment proteins as candidates for environmental perturbation by lead. Environ Health Perspect.

[b39-ehp-117-26] Six KM, Goyer RA (1970). Experimental enhancement of lead toxicity by low dietary calcium. J Lab Clin Med.

[b40-ehp-117-26] Sowers M, Jannausch M, Scholl T, Li W, Kemp FW, Bogden JD (2002). Blood lead concentrations and pregnancy outcomes. Arch Environ Health.

[b41-ehp-117-26] Téllez-Rojo MM, Hernández-Avila M, Lamadrid-Figueroa H, Smith D, Hernández-Cadena L, Mercado A (2004). Impact of bone lead and bone resorption on plasma and whole blood lead levels during pregnancy. Am J Epidemiol.

[b42-ehp-117-26] Willett WC, Reynolds RD, Cottrell-Hoehner S, Sampson L, Browne ML (1987). Validation of a semi-quantitative food frequency questionnaire: comparison with a 1-year diet record. J Am Diet Assoc.

[b43-ehp-117-26] Willett WC, Sampson L, Stampfer MJ, Rosner B, Bain C, Witschi J (1985). Reproducibility and validity of a semiquantitative food frequency questionnaire. Am J Epidemiol.

